# Exposure to artemether-lumefantrine (Coartem®) in first trimester pregnancy in an observational study in Zambia

**DOI:** 10.1186/s12936-015-0578-6

**Published:** 2015-02-14

**Authors:** Christine Manyando, Eric M Njunju, Mailis Virtanen, Kamal Hamed, Melba Gomes, Jean-Pierre Van geertruyden

**Affiliations:** Tropical Diseases Research Centre, Ndola, Zambia; International Health Unit, University of Antwerp, Antwerp, Belgium; Former Clinical Trial Leader, Novartis Pharma AG, Basel, Switzerland; Novartis Pharmaceuticals Corporation, East Hanover, NJ USA; World Health Organization/TDR, Geneva, Switzerland

## Abstract

**Background:**

In general, safety data following exposure to drugs in the first trimester of pregnancy are scarce. More specifically, data on the safety of artemisinin-based combination therapy (ACT) in pregnancy still remain limited. Therefore, pregnant women from Choma, Zambia, who were exposed to artemether-lumefantrine (AL) for the treatment of uncomplicated malaria were followed up and evaluated in a prospective cohort study. This report assessed the longitudinal safety outcomes of the pregnant women inadvertently exposed during the first trimester.

**Methods:**

Participants were classified based on the drug used to treat their most recent malaria episode: artemether-lumefantrine (AL) *versus* sulphadoxine-pyrimethamine (SP) and/or quinine. All enrolled women were followed up until six weeks post-delivery and the live births for 12 months.

**Results:**

There were 294 first trimester exposures in the observational cohort (pregnant women: AL = 150, AL and SP = 9 and SP and/or quinine = 135). Similar rates of perinatal mortality (stillbirths and neonatal deaths) were observed for each treatment arm (AL 4.4%, SP and/or quinine 3.9%). At delivery (newborns: AL = 135, AL and SP = 7 and SP and/or quinine = 129), the gestational age (measured using the Dubowitz total scores), length and head circumference of the newborns were similar between the two arms. Low birth weights were reported in 10.2% (95% CI 6.0, 16.6) and 6.7% (95% CI 3.4, 12.6) of newborns in the AL and SP and/or quinine arms, respectively. Overall development (including neurodevelopmental parameters) was similar between the two arms, both at 14 weeks and 12 months of age.

**Conclusion:**

Exposure to AL and SP in the first trimester was not associated with particular safety risks such as perinatal mortality, preterm deliveries or low birth weights. Such outcomes as well as infant neurodevelopmental parameters up to 12 months were similar between the two arms. These findings add to the body of data suggesting that randomized clinical trials could now be the way forward to assess safety and efficacy of ACT in the first trimester of pregnancy.

## Background

Women of childbearing age are at increased risk of malaria during pregnancy and the consequences of infection can be significant for them and for the fetus [1-3]. Although all malaria infections during pregnancy can affect maternal and foetal health, *Plasmodium falciparum* infection is associated with the most severe effects and has been linked to increased maternal, foetal and neonatal morbidity and mortality [3-5]. In areas of stable malaria transmission, asymptomatic malaria in pregnancy is associated with severe maternal anaemia, placental parasitaemia and low birth weight – a risk factor for death [6,7]. In areas of unstable transmission, pregnant women are at increased risk of severe malaria [3,7]. Therefore, it is important to ensure that effective prevention measures and treatments reach pregnant women in malaria endemic areas [6,8,9].

The World Health Organization (WHO) recommends the use of preventive measures to limit the occurrence of malaria in pregnancy, including the use of insecticide-treated nets (ITNs) in all malarious areas and intermittent preventive treatment (IPTp) in areas of stable malaria transmission. It also recommends that all cases of malaria during pregnancy should be treated promptly with an effective anti-malarial drug [10,11].

In the general non-pregnant population, artemisinin-based combination therapy (ACT) is the currently recommended first-line treatment for uncomplicated *P. falciparum* malaria and is known to be effective and well tolerated [12]. Risk-benefit data from 1,500 documented pregnancies exposed to artemisinin during the second or third trimester enabled the WHO to recommend the use of ACT as a first-line therapy to treat uncomplicated *P. falciparum* malaria during the second and third trimesters of pregnancy [13]. The recommended ACT for treatment includes artemether-lumefantrine (AL), artesunate-amodiaquine (AS-AQ), artesunate-mefloquine (AS-MQ) and artesunate-sulphadoxine-pyrimethamine (AS+SP), but excludes dihydroartemisinin-piperaquine (DHA-PPQ) because of lack of sufficient data for use of this combination in the second and third trimesters [13].

ACT is not recommended during the first trimester due to limited clinical safety data [13] and evidence of embryo lethality and some developmental abnormalities in animal studies [14,15]. WHO recommends a 7-day course of quinine plus clindamycin (or quinine monotherapy if clindamycin is not available) as the first-line malaria treatment in the first trimester, although there are concerns regarding poor tolerability (and compliance) related to quinine. Artesunate plus clindamycin is the recommended second-line treatment [13]. Other artemisinin-based combinations are indicated only if the recommended treatments are not available or have failed.

The necessary exclusion of pregnant women from clinical trials, including the original studies of artemisinins and ACT, and dependence upon observational studies explain the scarcity of data in this vulnerable population [16]. Consequently, monitoring the safety of ACT in first-trimester pregnancy still remains an important area of malaria research. An understanding of the risks and benefits of ACT during early pregnancy is needed to guide policymakers and healthcare workers, and ensure that pregnant women receive both effective and safe treatments for malaria [16]. A population-based study carried out in Thailand with over 17,000 participants including 945 women who had malaria in the first trimester reported that first trimester exposure to artesunate showed no significant difference in birth outcomes as compared to quinine or chloroquine [17]. Treatment related adverse effects were not seen, although the number of women treated with artesunate was small [17].

In Zambia, AL is the first-line drug for treatment of malaria in the general population. As per WHO guidelines, it is contraindicated in the first trimester of pregnancy. However, this anti-malarial is sometimes used in practice during the first trimester, because women often do not disclose their pregnancy in the early stages for cultural reasons. In a previously reported observational study conducted in Zambia from 2004 to 2008 [18], some women were inadvertently exposed to AL in the first trimester of pregnancy. The safety data from these exposures to AL or SP and/or quinine in the first trimester of pregnancy form the basis of this report.

## Methods

### Study population

The first trimester exposed pregnant women were part of the observational cohort study that was carried out in Zambia at four antenatal clinics in the districts of Choma, Ndola and Lusaka from October 2004 to July 2008 [18]. These women were eligible for inclusion if they had received AL or SP and/or quinine for the treatment of uncomplicated malaria. Diagnosis was clinically or parasitologically confirmed. At the time, SP was the standard anti-malarial treatment during pregnancy, and was also used for IPTp. Pregnant women were assigned to treatment arms according to the treatment received for the “index episode”, defined as the most recently treated malaria episode prior to study entry.

### Study procedures

Data from women who had inadvertent first trimester exposure to AL were analysed descriptively to compare outcomes between first trimester and second and third trimester exposures for the following (first trimester) subgroups: exposure to AL alone; exposure to AL plus other anti-malarials; exposure to anti-malarial(s) other than AL; no anti-malarial drug exposure; and unknown anti-malarial drug exposure (due to missing or incomplete last menstrual period [LMP] date).

The primary endpoint for the prospective cohort was the incidence of perinatal mortality (stillbirth or neonatal death within seven days of birth). Secondary endpoints were gestational age at delivery and birth weight adjusted for gestational age [19]. A newborn was considered to have low birth weight if the observed birth weight was lower than the corresponding 5th percentile of the Zimbabwean birth weight according to gestational age, in keeping with international standards. Exploratory measures that were assessed included frequency of spontaneous abortion, preterm delivery, neonatal mortality, maternal mortality, major and minor birth defects, and infant development including neurological development.

Women attending antenatal clinics were enrolled if they reported receiving treatment with either AL (20 mg artemether plus 120 mg lumefantrine) or SP (1500 mg sulphadoxine plus 75 mg pyrimethamine) and/or quinine according to label recommendations. Exposure was verified by documentation from outpatient clinic files, which also detailed any diagnostic procedures performed and concomitant medications given. As per government policy, women received SP, at the doses described above, during the second or third trimester as IPTp. Treatment with other drugs, including anti-malarials, prior to or after the index episode was recorded [18].

First trimester pregnancies were defined as pregnancies with a gestational age of less than 14 weeks. Women visited the antenatal clinics for assessment of safety parameters at baseline/enrolment, 4 weeks post-enrolment, 4 weeks pre-delivery, at delivery, and at 6 weeks post-delivery. Infants were followed up at 6 weeks, 14 weeks and 12 months after birth.

At delivery, the newborn’s vital signs and APGAR score at one minute, five minutes and 10 minutes after birth were recorded. Birth weight was measured in grams using a digital scale with a precision of two decimal points. At the antenatal care clinics, study participants were encouraged to deliver at the health facility by the study midwives. For deliveries outside of the health facility, birth weight was measured at a health facility within 24 hours after birth if such a visit occurred. Quality control measures included monthly calibration of the baby scales using standard pre-measured commercially purchased weights. The gestational age was estimated from the date of the LMP and was confirmed using the Dubowitz neurological and external criteria for gestational age assessment at birth. The study midwives who performed these assessments were trained by the study paediatrician. Detection of major and minor birth defects was performed by study nurse/midwives in accordance with the data collection form (checklist) at birth and at 6 weeks post-delivery in home delivery cases. The study physicians re-assessed babies with congenital malformations to confirm findings. They also periodically and randomly verified measurements of head circumference and length. In addition, the study paediatrician visited the health facilities periodically to review gestational age assessments and verified congenital malformations.

Safety assessments included monitoring and recording of all adverse events (AEs) and serious adverse events (SAEs) up to six weeks post-delivery. Pregnancy-specific assessments included rates of perinatal mortality defined as stillbirth (>28 weeks gestation) and early neonatal death (within seven days of birth); neonatal mortality (≤28 days of birth); maternal mortality (up to six weeks post-delivery); spontaneous abortion (≤28 weeks gestation); stillbirth; preterm delivery (≤37 completed weeks); incidence of low birth weight; gestational age at delivery (estimated from LMP, or by a Dubowitz score [20,21] if the LMP was unknown); and incidence of major and minor birth defects. Concomitant infections were recorded. Both major and minor birth malformations were documented using a checklist [18].

Neurodevelopmental assessment was performed by the investigators at 14 weeks and 12 months after birth, either through a general assessment (e.g. smiling, lifting head, sitting unsupported, standing without assistance, and crawling) or the Shoklo neurodevelopment assessment [22], or both.

This study was designed, implemented, and reported in accordance with ICH Good Clinical Practice, applicable local regulations, and the Declaration of Helsinki. The study protocol was first approved by the Institutional Review Boards at Tropical Diseases Research Centre, Zambia (TDRC) and WHO/TDR in Geneva before approval by the local Ethics Review Committee of the TDRC, and WHO Ethics Review Committee, Geneva. All participants, or their parent/guardian (if the subject was a minor), gave written or finger-marked informed consent before study entry. An independent Study Advisory Committee reviewed unblinded data for the whole cohort, including the first trimester exposures during and at the end of the study.

## Results

This report concentrates on first trimester exposures as analyses of the full cohort were previously published [18]; as a result there is some overlap with published results. Women with first trimester exposures had similar baseline characteristics to the overall cohort. Over 95% attained primary education and over 52% attained secondary education. Alcohol intake was similar between the treatment arms, not exceeding 3%. None of the women were smokers. Age ranges and weight profiles across the treatment groups were similar (Table 1).

**Table 1:** **Age, body weight at enrolment and treatment courses during first trimester including IPTp with SP**

**Exposure group**					
	**AL, N=150**	**AL** ^*^ **and SP** ^**†**^ **, N=9**	**SP** ^**†**^ **and/or quinine, N=135**	**No anti-malarial, N=667**	**Unknown anti-malarial, N=40**
**Age group, n (%)**					
<20 years	20 (13.3)	1 (11.1)	14 (10.4)	118 (17.7)	7 (17.5)
20-24 years	56 (37.3)	5 (55.6)	41 (30.4)	204 (30.6)	12 (30.0)
25-29 years	35 (23.3)	2 (22.2)	44 (32.6)	199 (29.8)	12 (30.0)
30-39 years	36 (24.0)	1 (11.1)	35 (25.9)	139 (20.8)	9 (22.5)
>40 years	3 (2.0)	0 (0)	1 (0.7)	7 (1.1)	0 (0)
**Body weight (kg)**					
Mean (± SD)	61.2 (±9.8)	60.3 (±10.1)	60.8 (±10.0)	60.3 (±10.1)	60.8 (±10.0)
Median	60.0	59.0	60.0	59.0	59.7

AL = artemether-lumefantrine; SP = sulphadoxine-pyrimethamine.

^*^AL treatment in first trimester; ^†^SP treatment in first trimester.

Exposure groups represent the treatment given to the pregnant woman for the malaria episode that had occurred most recently prior to enrolment (= index episode).

**Figure 1:**
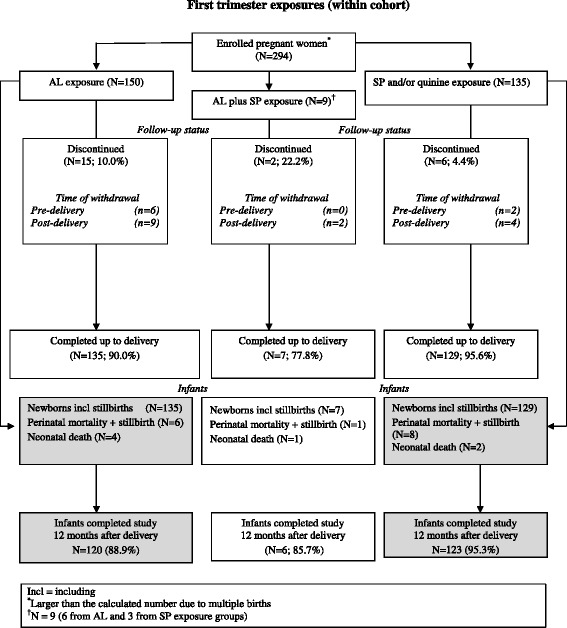
**First trimester exposures (within cohort).**

The study population was selected based on treatment received for the most recent (index) episode of uncomplicated malaria prior to enrolment. The index malaria episode was predominantly diagnosed based on symptoms. In total, 294 women out of the cohort of 1,001 (29.4%) received treatment in the first trimester of pregnancy. Of these, 156 women (150/495 (30.3%)) from the AL group, plus six from the SP group) received AL treatment during the first trimester, while 138 women (135/506 (26.7%)) from the SP and/or quinine group, plus three from the AL group) received SP treatment during the first trimester (Figure 1). Women with first trimester anti-malarial treatment also received concomitant medications such as haematinics (oral iron preparations and folic acid).

Despite counselling of all enrolled women on human immunodeficiency virus (HIV) testing, only 30% in the AL arm and 38% in the SP arm agreed to be tested; 7.2% of those with a test result were HIV positive (AL 7.3%, SP and/or quinine 7.1%). Respiratory tract infections were reported in 4% and 5% of women in the AL and SP and/or quinine arms, respectively. Other infections (e.g. urinary tract infections, syphilis) were recorded less frequently. Non-infection-related medical conditions were recorded in ≤2% of women in either arm.

As reported previously [18], patients exposed only to AL during the first trimester had similar rates of preterm and full term deliveries to patients who did not receive anti-malarials during the first trimester; 86% of these women had normal deliveries (Table 2). A small proportion had caesarean sections (AL 2.7%, SP and/or quinine 3.0%).

**Table 2:** **Delivery outcomes by anti-malarial drug exposure during first trimester**

	**Exposure group**				
**Type of delivery**	**AL only, N=135, n (%)**	**AL and SP, N=7, n (%)**	**SP and/or quinine, N=129, n (%)**	**No anti-malarial, N=644, n (%)**	**Unknown anti-malarial, N=40, n (%)**
Normal	129 (86.0)	6 (66.7)	125 (92.6)	623 (91.1)	39 (92.9)
Caesarian section	4 (2.7)	0 (0)	4 (3.0)	21 (3.1)	1 (2.4)
Other (instrumental deliveries)	2 (1.3)	1 (11.1)	0 (0)	0 (0)	0 (0)

Birth weights and birth weights adjusted for gestational age by exposure group were generally similar (low birth weights: AL 10.2% (95% CI 6.0, 16.6), SP and/or quinine 6.7% (95% CI 3.4, 12.6) and no anti-malarials 8.7% (95% CI 6.7, 11.2)). There were no major differences between newborns of women who were exposed to AL only, anti-malarials other than AL (SP and/or quinine), or no anti-malarials during first trimester (Table 3).

**Table 3:** **Newborn birth weight and birth weight adjusted for gestational age by exposure group, according to anti-malarial drug exposure during first trimester**

	**Exposure group**				
	**AL only, N=133**	**AL and SP, N=7**	**SP and/or quinine, N=126**	**No anti-malarial, N=627**	**Unknown anti-malarial, N=40**
	**Birth weight (g)**				
N	128	7	120	597	40
Mean	3043	3010	3065	3046	3066
Standard deviation	560	690	466	501	503
	**Birth weight (g) adjusted for gestational age, n (%)** [19]				
Low	13 (10.2)	1 (14.3)	8 (6.7)	52 (8.7)	0 (0)
Normal	115 (89.8)	6 (85.7)	112 (93.3)	545 (91.3)	34 (100.0)
Missing	5	0	6	30	6

Birth weights from singleton live births only, measured within 24 hours of delivery.

The assessments performed at birth such as gestational age calculated from total Dubowitz scores, newborn length and head circumference showed no major differences across the exposure groups (Table 4). These are not depicted according to international standards for reporting by gestational age and sex due to lack of Zambian references for newborn measurements of weight, length and head circumference [23].

**Table 4:** **Newborn gestational age, length and head circumference, according to anti-malarial drug exposure during first trimester**

	**Exposure group**				
	**AL only, N=133**	**AL and SP, N=7**	**SP and/or quinine, N=126**	**No anti-malarial, N=627**	**Unknown anti-malarial, N=40**
	**Gestational age (weeks)**				
Minimum	28.8	28.8	28.8	28.8	17.0
25th Quartile	36.2	36.2	37.0	36.5	36.2
Median	37.7	37.7	37.7	37.8	38.1
75th Quartile	38.9	38.9	39.6	38.9	38.9
Maximum	41.8	41.8	42.0	41.8	41.8
	**Length (cm)**				
Minimum	27.0	30.0	34.0	30.0	31.0
25th Quartile	48.0	48.0	47.0	48.0	47.0
Median	49.0	49.4	49.0	49.0	49.0
75th Quartile	50.0	50.0	50.0	50.0	50.0
Maximum	60.0	60.0	59.0	60.0	59.0
	**Head circumference (cm)**				
Minimum	24.0	24.0	24.0	24.0	24.5
25th Quartile	34.0	34.0	34.0	34.0	34.0
Median	36.0	35.4	35.0	36.0	35.0
75th Quartile	37.0	36.0	36.0	37.0	36.0
Maximum	41.0	43.0	43.0	41.0	44.0

There were generally no major differences across the exposure groups in adverse birth outcomes among the women with complications. There were more premature births among the babies born to women exposed to SP and/or quinine (23%; 95% CI 16.7, 30.7) than those exposed to AL (16.7%; 95% CI 11.6, 23.5), but the difference was not statistically significant. There were no other differences between the arms.

Table 5 disaggregates perinatal and neonatal deaths by time. Perinatal mortality outcomes were similar for the AL group (4.4%; 95% CI 2.1, 9.4), the SP and/or quinine group (3.9%; 95% CI 1.7, 8.8) and those who had no anti-malarial exposure in first trimester (4.8%; 95% CI 3.4, 6.8). Early neonatal deaths (i.e. within 28 days) were also similar for the AL exposure group (3.8%; 95% CI 1.6, 8.5) as compared with those who had no anti-malarial exposure in the first trimester (3.0%; 95% CI 1.9, 4.7).

**Table 5:** **Perinatal and neonatal mortality by anti-malarial drug exposure during first trimester**

	**Exposure group**				
Outcome of deliveries (N*)	**AL only, N=135, n (%), (95% CI)**	**AL and SP, N=7, n (%), (95% CI)**	**SP and/or quinine, N=129, n (%), (95% CI)**	**No anti-malarial, N=644, n (%), (95% CI)**	**Unknown anti-malarial, N=40, n (%), (95% CI)**
Perinatal mortality, n (%)	6 (4.4)	1 (14.3)	5 (3.9)	31 (4.8)	1 (2.5)
	(2.1, 9.4)	(2.6. 51.3)	(1.7, 8.8)	(3.4, 6.8)	(0.4, 12.9)
Stillbirth (>28 weeks gestation), n (%)	2 (1.5)	0 (0)	3 (2.3)	17 (2.6)	0 (0)
	(0.4, 5.2)	(0.0, 39.0)	(0.8, 6.6)	(1.7, 4.2)	(0.0, 9.0)
Death ≤7 days after birth, n (%)	4 (3.0)	1 (14.3)	2 (1.6)	14 (2.2)	1 (2.5)
	(1.2, 7.4)	(2.6, 51.3)	(0.4, 5.5)	(1.3, 3.6)	(0.4, 12.9)
	**Exposure group**				
**Outcome of neonates (N** ^**†**^ **)**	**AL only, N=133, n (%), (95% CI)**	**AL and SP, N=7, n (%), (95% CI)**	**SP and/or quinine, N=126, n (%), (95% CI)**	**No anti-malarial, N=627, n (%), (95% CI)**	**Unknown anti-malarial, N=40, n (%), (95% CI)**
Neonatal mortality within 28 days of birth, n (%)	5 (3.8)	1 (14.3)	2 (1.6)	19 (3.0)	1 (2.5)
	(1.6, 8.5)	(2.6, 51.3)	(0.4, 5.6)	(1.9, 4.7)	(0.4, 12.9)
Infant mortality >28 days to 1 year afer birth^‡^ n (%)	8 (6.3)	0 (0.0)	1 (0.8)	21 (3.5)	0 (0.0)
	(3.2, 11.9)	(0.0, 39.0)	(0.1, 4.4)	(2.3, 5.2)	(0.0, 9.0)
Total infant mortality within 1 year after birth, n (%)	13 (9.8)	1 (14.3)	3 (2.4)	40 (6.4)	1 (2.5)
	(5.8, 16.0)	(2.6, 51.3)	(0.8, 6.8)	(4.7, 8.6)	(0.4, 12.9)

Perinatal mortality: stillbirth or early neonatal death within 7 days after birth; CI = confidence interval.

^*^All deliveries; ^†^Total live births; ^‡^Denominators: AL only N=128; AL and SP N=6; SP and/or quinine N=124; No anti-malarial N=608; and Unknown anti-malarial N=39.

The number of malformations in 130 babies born to mothers who received only AL in the first trimester was 9 (6.9%), and this frequency was not different from that of patients exposed to other anti-malarials (8/121, 6.6%). In all but one case of exposure only to AL, the malformations were umbilical hernias. For all infants with umbilical hernia, the hernia was not detected at birth, but at week 6 (6 cases) or week 14 (2 cases) visits post-delivery.

Neurodevelopmental assessment of infants from the Choma site using the Shoklo development test is summarized according to anti-malarial exposure in the first trimester in Table 6. The evaluation was carried out at 14 weeks and 12 months after birth. The results of the assessment appeared to be similar across all exposure groups. At 14 weeks, 99% of infants of mothers who received only AL during the first trimester had assessments rated ‘good’ or ‘excellent’, as did 100% at 12 months. There was no difference in scores for coordination, tone, behaviour and overall milestones between the treatment arms at 14 weeks and 12 months after birth. Results were similar for infants of patients who received other anti-malarials, or no anti-malarials, during the first trimester.

**Table 6:** **Neurodevelopmental assessment according to anti-malarial drug exposure during first trimester**

	**Exposure group**				
	**AL only**	**AL and SP**	**SP and/or quinine**	**No anti-malarial**	**Unknown anti-malarial**
**14 weeks after delivery**	**N=81, Median (range)**	**N=3, Median (range)**	**N=48, Median (range)**	**N=367, Median (range)**	**N=22, Median (range)**
Motor milestones	9.0	12.0	7.0	9.0	11.5
	(6.0-18.0)	(12.0-13.0)	(5.0-37.0)	(2.0-18.0)	(6.0-24.0)
Coordination	5.0	10.0	5.0	6.0	6.0
	(4.0-20.0)	(5.0-11.0)	(4.0-34.0)	(2.0-21.0)	(6.0-6.0)
Tone	18.0	18.0	18.0	18.0	17.5
	(15.0-23.0)	(17.0-19.0)	(16.0-20.0)	(13.0-24.0)	(14.0-23.0)
Behaviour	15.0	15.0	15.0	15.0	15
	(12.0-15.0)	(15.0-15.0)	(10.0-15.0)	(10.0-15.0)	(12.0-15.0)
**14 weeks after delivery**	**N=81, Mean (SD)**	**N=3, Mean (SD)**	**N=48, Mean (SD)**	**N=367, Mean (SD)**	**N=22, Mean (SD)**
Height (cm)	59.9 (4.11)	59.6 (4.13)	59.2 (4.17)	59.6 (4.15)	59.8 (4.12)
Weight (kg)	6.7 (0.94)	6.6 (0.99)	6.6 (0.93)	6.6 (0.93)	6.6 (0.98)
**One year after delivery**	**N=75, Median (range)**	**N=3, Median (range)**	**N=49, Median (range)**	**N=365, Median (range)**	**N=20, Median (range)**
Motor milestones	35.0	35.0	34.0	35.0	35.0
	(25.0-37.0)	(31.0-37.0)	(25.0-37.0)	(20.0-37.0)	(28.0-37.0)
Coordination	32.0	26.0	32.0	32.0	31.0
	(19.0-34.0)	(26.0-32.0)	(5.0-33.0)	(12.0-34.0)	(21.0-33.0)
Tone	20.0	18.0	19.0	20.0	18.0
	(16.0-20.0)	(17.0-20.0)	(16.0-20.0)	(15.0-24.0)	(16.0-24.0)
Behaviour	15.0	15.0	15.0	15.0	15.0
	(12.0-15.0)	(15.0-15.0)	(7.0-15.0)	(9.0-15.0)	(13.0-15.0)
**One year after delivery**	**N=75, Mean (SD)**	**N=3, Mean (SD)**	**N=49, Mean (SD)**	**N=365, Mean (SD)**	**N=20, Mean (SD)**
Height (cm)	70.5 (6.46)	69.0 (6.32)	68.9 (6.22)	70.0 (6.43)	69.0 (6.35)
Weight (kg)	9.5 (1.08)	9.4 (1.08)	9.4 (1.09)	9.3 (1.09)	9.4 (1.08)

Neurodevelopmental assessment done in only 60% of all study participants.

## Discussion

Although not recommended for use in the first trimester of pregnancy, a substantial proportion of patients were exposed to AL during this period. As it is a cultural norm in Zambia not to disclose a pregnancy before it is visible, it is probable that some women intentionally did not disclose their pregnancies to their healthcare providers. Alternatively, some women may have been pregnant and were prescribed AL before they were aware of their status as it is not routine practice to perform a pregnancy test before administration of medications to women of childbearing ages at outpatient clinics. As a consequence of these unexpectedly high exposure rates within the first few months after initiation of the observational cohort, the Central Board of Health (CBoH) was persuaded to re-issue guidelines and distribute them to all health institutions in the country emphasizing the contraindication of AL in the first trimester.

Nevertheless, first trimester exposure to AL was not associated with a greater risk of perinatal or neonatal death or stillbirths even among women with complications at delivery. However, there were more patients in the AL exposure group with spontaneous abortions, albeit with additional first trimester risk factors (including malaria or another febrile illness) associated with miscarriage [17]. Unfortunately, women who had febrile illnesses presumed to be malaria had no parasitological confirmation of malaria.

Though drug safety concerns still restrict the recommended treatment for *P. falciparum* malaria in the first trimester to a seven-day treatment with quinine, a poorly tolerated, poorly adhered to, and therefore less effective treatment testifies to the role malaria or other febrile illnesses play in contributing to the adverse outcomes of pregnancy. This does not mean that AL did not cause or contribute to adverse pregnancy outcomes, but rather that it is difficult to disentangle the effects of malaria from those of its treatment, particularly in women who may not yet know that they are pregnant. Therefore, further studies to explore the safety and efficacy of anti-malarials, which could be used in early pregnancy, are needed. Establishing the safety of artemisinins in early pregnancy is an important area of research. In the very large cohort from Thailand, exposure to artemisinin derivatives was not associated with miscarriage after controlling for maternal and malaria characteristics [17], and birth outcomes did not differ significantly after first trimester treatment with artesunate compared with other treatments, such as quinine and chloroquine.

In the full cohort analysis [18], the profile of AEs reported was unremarkable. Except for complications of pregnancy, most of the commonly reported AEs were related to malaria or other infections [18]. The great majority of the SAEs reported were related to complications of pregnancy, mostly presenting as premature delivery. Other SAEs were very infrequent and were mainly infections.

The study has some limitations. First, patients entered the study after completing treatment for the index episode of malaria, and this made the tolerability of treatment difficult to assess. Therefore, these safety data reflect pregnancy-related outcomes in a malaria endemic area of Zambia, including IPTp administration, rather than AEs related to the treatment of an index malaria episode. Furthermore, in 82% of the AL and 87.2% of the SP patients, the index episode of malaria was unconfirmed either by microscopy or by rapid diagnostic test, and it is therefore possible that some patients had febrile illnesses other than malaria. Parasitological information would have helped to better interpret the findings. Failure to test for malaria was routine practice at the time of the study. Today, following WHO treatment guidelines and NMCP policy, ACT treatment of all patients, including women of childbearing age, is only provided after parasitological testing.

It was only possible to crudely estimate gestational age using LMP as ultrasound was unavailable routinely. Therefore, it is possible that some infants may have been classified as premature when in reality they were not. Nevertheless, there are recent data indicating that LMP may be a more reliable method for measuring gestational age than previously thought [24].

Finally, due to lack of consenting for HIV testing by the majority of women in the study, the rate of HIV infection may have been considerably higher than 7%. Reported rates of HIV infection in women attending antenatal clinics in Zambia in 2002 ranged from 11% in rural areas to 26% in urban areas [25].

## Conclusion

Exposure to AL in the first trimester was not associated with higher risk of perinatal or neonatal mortality, premature delivery or low birth weight compared with SP. Although limited, the available data add to the evidence on the safety of AL and artemisinins in general during first trimester pregnancy. Should evidence continue to be reassuring, randomized clinical trials might be considered to reliably determine the safety and efficacy of ACT in the first trimester of pregnancy.
